# Persistence of Structural Lubricity on Contaminated Graphite: Rejuvenation, Aging, and Friction Switches

**DOI:** 10.1021/acs.nanolett.4c02883

**Published:** 2024-09-23

**Authors:** Wai H. Oo, Hongyu Gao, Martin H. Müser, Mehmet Z. Baykara

**Affiliations:** † Department of Mechanical Engineering, University of California Merced, Merced, California 95343, United States; ‡ Department of Materials Science and Engineering, Saarland University, Saarbrücken, Saarland 66123, Germany

**Keywords:** Atomic force microscopy, Friction mechanisms, Gold nanoislands, Molecular dynamics, Structural lubricity, Surface contamination

## Abstract

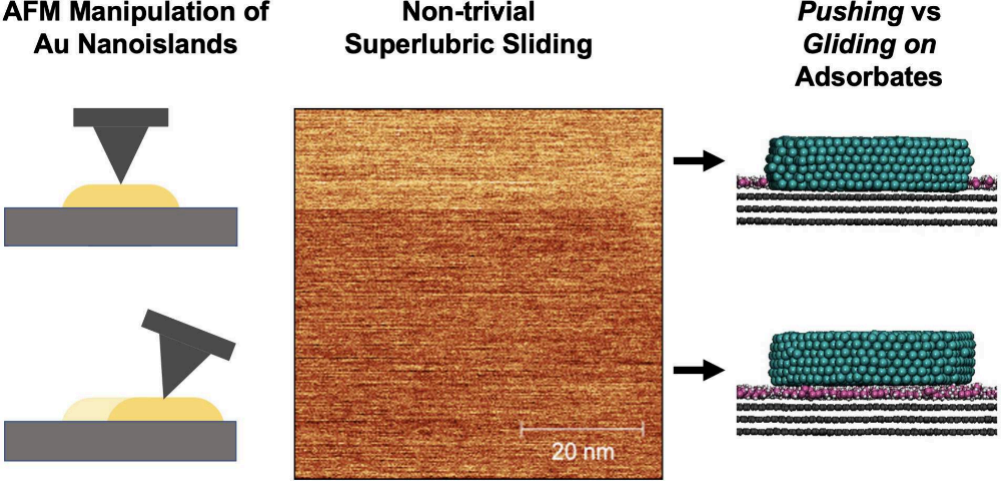

Using atomic force microscopy experiments and molecular dynamics simulations of gold nanoislands on graphite, we investigate why ultralow friction commonly associated with structural lubricity can be observed even under ambient conditions. Measurements conducted within a few days after sample synthesis reveal previously undiscovered phenomena in structurally lubric systems: *rejuvenation*, a drop in kinetic friction of an order of magnitude shortly after the onset of sliding; *aging*, a significant increase in kinetic friction forces after a rest period of 30 min or more; and *switches*, spontaneous jumps between distinct friction branches. These three effects are drastically suppressed a few weeks later. Imaging of a contamination layer and simulations provide a consistent picture of how single- and double-layer contamination underneath the gold nanoislands as well as contamination surrounding the nanoislands affect structural lubricity without leading to its breakdown.

Research on structural lubricity, an ultralow friction state arising due to the systematic annihilation of lateral forces in atomically flat interfaces formed by two incommensurate surfaces,^1−3^ is accelerating in recent years.^4,5^ Although the concept came about as a theoretical exercise,^1,2^ atomic force microscopy (AFM) experiments on the friction between a graphene flake and graphite under dry nitrogen atmosphere confirmed the geometrical explanation of miniscule friction due to rotation-induced lattice mismatch.^6^ More recently, other observations of ultralow friction attributed to structural lubricity between carbon-based materials have been reported,^7,8^ complemented by reports of structural lubricity at heterointerfaces formed by two-dimensional materials.^9,10^ Likewise, theoretical predictions of surface contamination leading to a breakdown of structural lubricity^11^ were supported through AFM experiments performed on antimony nanoislands (for simplicity, “island” will be used in place of “nanoisland” hereafter) on graphite, contrasting ultrahigh vacuum (UHV) with ambient conditions.^12^ Remarkably, similar experiments performed on noble metal islands on graphite demonstrated that structural lubricity is not restricted to the pristine UHV environment. Comparable values of friction force and the sublinear scaling of friction force as a function of contact area were also observed under uncontrolled ambient conditions.^13,14^ This raises the questions of when contaminants destroy structural lubricity and how they affect friction otherwise, which is relevant for the potential exploitation of superlubricity outside of UHV chambers. Contaminating interfacial particles were expected to destroy superlubricity because their mobility allows them to adopt positions, which are energy minima of both surfaces simultaneously, whereby the surfaces interlock.^11^ One reason this argument may not always hold is that extremely smooth surfaces like that of graphite do not provide energy barriers large enough to substantially counteract sliding of an adsorbed or rather “between-sorbed” layer. Another one is that organic molecules adsorbed on graphite form highly ordered domains.^15^ They might act similarly to two-dimensional solids, whereby individual molecules have an additional constraint in the presence of another surface, which reestablishes, to a significant degree, the systematic annihilation of lateral forces at pristine interfaces formed by smooth, incommensurate surfaces. Which effect dominates might depend on the structure and chemical nature of the counterface to graphite.

To illuminate the role of contamination in superlubricity, we present results of AFM-based sliding (i.e., nanomanipulation) experiments on gold islands. In contrast to other experiments on this system, we chose the tip-on-top^16^ rather than the previously used and easier-to-implement push-from-the-side approach,^13,14^ because it allows islands to be dragged back and forth under a controlled load in a controlled direction, with controlled speeds. We also report results of molecular dynamics (MD) simulations mimicking the experiments, albeit using smaller islands and nine-order-of-magnitude larger velocities. Thus, direct quantitative comparisons are not possible and would remain questionable even when using scaling arguments. Nonetheless, relative trends of how contamination affects friction can certainly be contrasted, whereby MD can offer possible explanations for experimental observations.

Before presenting the results of the nanomanipulation experiments regarding friction forces, we briefly describe the associated data acquisition procedure. First, individual line scans of lateral force, such as those shown in Figure 1d are recorded. In accordance with established procedures,^17^ half of the difference between trace (i.e., forward) and retrace (i.e., backward) scans are plotted for all lines (*n* = 1, 2, ..., 256), yielding maps of friction like those shown in Figure 1c,f. While some friction maps are homogeneous (Figure 1c), some reveal two clearly distinct domains of high and low friction, as indicated by bright and light colors, respectively (Figure 1f). Data points in the remaining panels of Figure 1 represent the average over such friction domains. Since no areal scan produced more than two domains, one scan can result in one (Figure 1a,b) or two (Figure 1e) friction values. Arrows in the plot shown in Figure 1e indicate the jumps from low to high friction domains.

**Figure 1:**
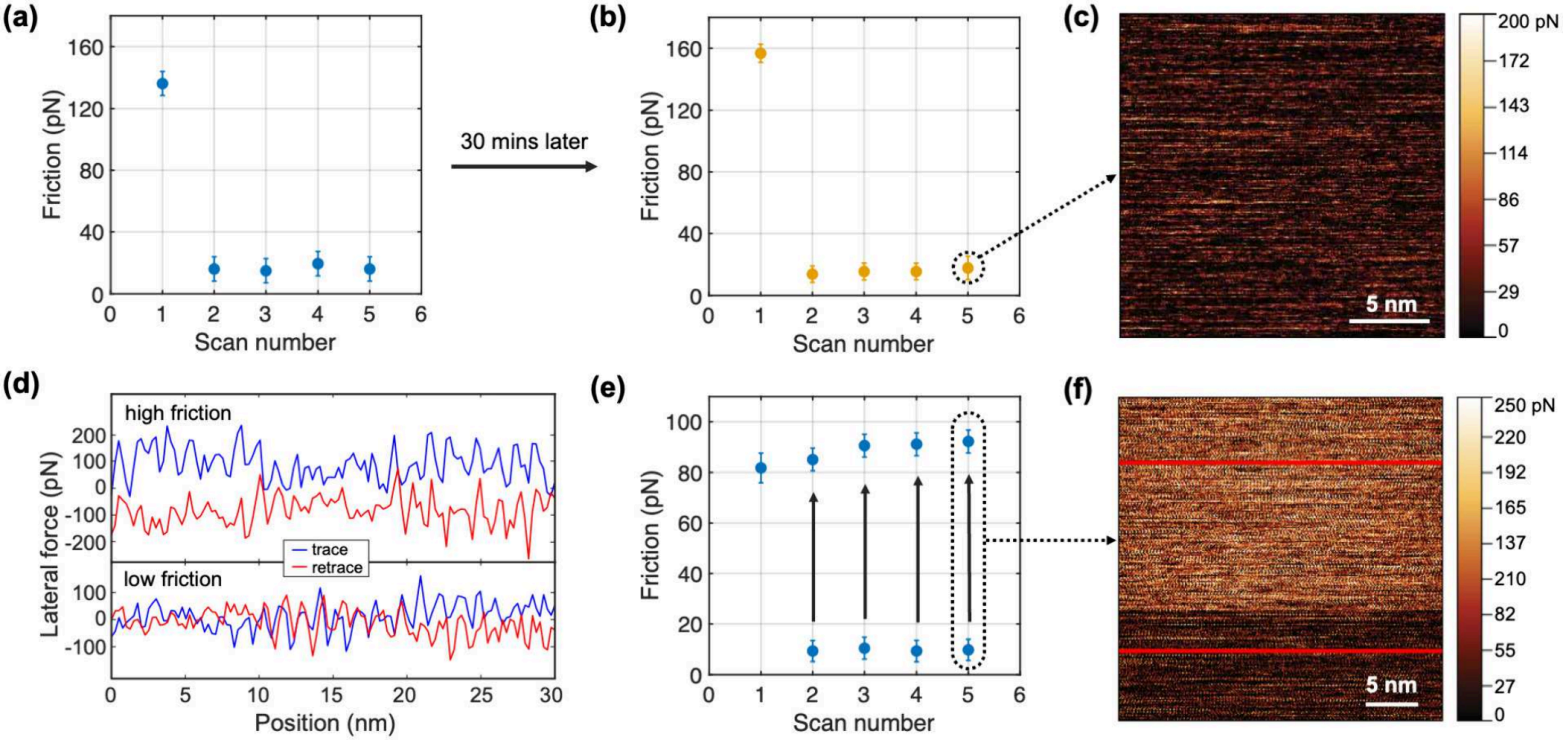
(a) Friction force as a function of areal scan number extracted from the nanomanipulation of a freshly deposited island, where “Scan number” refers to the order in which the images were recorded. (b) Same as (a) but repeated on the same island and area after a 30 min waiting time. (c) Friction-force map associated with scan 5 in (b). (d) Lateral forces extracted along single scan lines in the forward and backward directions, along the two traces shown with red lines in (f), located in high and low friction domains, respectively. (e) Friction force as a function of scan number extracted from the nanomanipulation of another gold island. During the scan, friction jumped from low to high, as highlighted by black arrows, and as shown in the friction-force map of panel (f). Error bars in panels (a), (b), and (e) represent standard error of the mean.

Gold islands, when measured within a few days after synthesis, always produce relatively high friction during the first areal scan. However, friction drops substantially during the second scan, i.e., by 1 order of magnitude and can remain low in all subsequent scans, as is depicted in Figure 1a. When repeating the experiments on the same island and same scan area, after waiting times ranging from 30 min to 18 h, similar results were obtained (Figure 1b). Differences between the average friction of the very initial scan and the first scan after ≥30 min waiting times were on the order of 15%. The average friction of subsequent scans were again reduced by about an order of magnitude with respect to the first one as in the initial set of experiments. Interestingly, stiction peaks were observed neither at the onset of a first scan (initial and after waiting) nor after the periodic pauses (on the order of 10 ms) at the end of scan lines, where the sliding direction changes.

An increase of friction force with waiting time has been known since Coulomb’s classical friction experiments^18^ and is commonly referred to as *aging*,^19^ while the reduction of friction during sliding is called *rejuvenation*(20) and captured in rate-and-state models of friction.^21,22^ However, typical results reveal changes on the order of 10% rather than by a factor of 10. Prominent examples are rough glassy polymer sliding past smooth silanized glass with nominal contact areas on the order of millimeters^20^ as well as material systems meant to mimic the friction of tectonic plates.^21,22^ Given the dramatic difference in the observed effect, common explanations of aging and rejuvenation^23^ do not seem plausible for our experiments.

In addition to rejuvenation and aging, we encountered “friction switches”, i.e., spontaneous *jumps* between low and high friction forces within a single scan, (see Figure 1e,f). A combination of all three effects is frequently observed within the same experiment, even with the same island on the same area (see Figure S5). Since graphite has a homogeneous surface away from steps and operating conditions are mild, surface contamination appears to be the most likely candidate to cause aging, rejuvenation, and friction switches in our system, which is why it is investigated next.

To gain information on contamination of our samples, we used AFM-based topographical and phase imaging. As opposed to topographical imaging, which solely provides height information, phase imaging allows materials with different mechanical stiffness on the sample surface to be distinguished with high spatial resolution.^24^Figure 2 shows a large-scale phase image taken via tapping-mode AFM several months after synthesis. It reveals dark regions indicating soft contaminants and light regions for graphite and gold islands. Surface contamination becomes apparent as early as 1 week postsynthesis, despite the samples having been stored in a vacuum desiccator with the intention to deter environmental adsorbates from accumulating on the surface. The measured height associated with contamination, as inferred from topographical AFM imaging (see Figure S6) ranges from a few Å up to 10 Å, which corresponds to 1–2 adsorbate layers.

**Figure 2:**
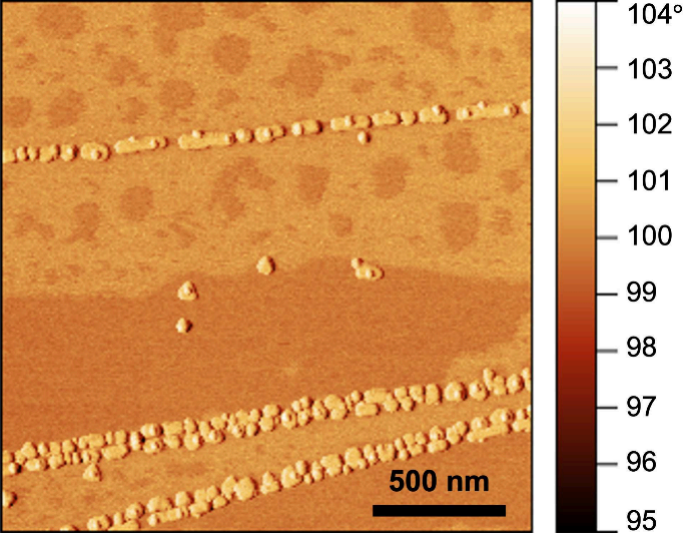
AFM phase image of the sample system, taken 4 months after synthesis, showing the accumulation of contaminant layers.

Manipulation of an island on a heavily contaminated sample causes a localized change in the substance’s coverage (Figure S6a,b). However, the contamination layer can also change over time in both coverage and morphology without nanomanipulation (Figure S6d). Upon heating the sample to 100 °C, AFM images have no more contrast (see Figure S7) implying that the contaminant is desorbed or has become very homogeneous, e.g., in the form of an alkane layer with alkanes having more than ≈20 carbon atoms.^25,26^ According to these characteristics, the initial contaminant layer is most plausibly a mixture of water and hydrocarbons. As revealed by a recent study, this combination is frequently found under ambient conditions on van der Waals materials, such as graphite and hBN.^27^ More specifically, it is proposed that the contaminant layer comprises predominantly midlength linear alkanes containing 20–26 backbone carbons.

Although thicker lubrication layers imply a reduced resistance to sliding in continuum mechanics and despite much knowledge about the slip of alkanes past graphitic^28^ and gold surfaces alike^29^ even under high-confinement conditions,^30^ friction in our system appears impossible to predict from the existing literature. This is because friction at the nanoscale often is a true system property,^23^ which cannot be deduced from those of the lubricant and its two boundaries with the confining walls. Details like the relative orientation of the confining walls can matter^11^ or confinement-induced structural transformation of the lubricant can occur, as might be the case for alkanes between gold.^29^ This motivated us to elucidate the dependence of shear stress on adsorbate coverage and other parameters for this particular system using molecular dynamics.

The simulations reveal a highly nonmonotonic dependence of friction on coverage between the simulated island and the graphite substrate, see Figure 3. It is almost immeasurably small at zero coverage due to the absence of any significant instabilities,^31^ as indicated by the blue, dashed line. The effective shear stress, i.e., the ratio of friction force and island area, is largest at submonolayer coverages (Γ) when the gold island rests directly on graphite but has to displace polymers in direct contact with graphite out of the way while advancing. Thus, in this case, friction stems predominantly from the out-of-contact rather than the contact region itself. Friction drops discontinuously upon increasing Γ, once the island no longer touches graphite directly, see Figure 3b. It also reveals the alkanes to orient along graphite’s symmetry axis, thereby forming locally commensurate domains, which explains why slip at full coverage occurs between gold and adsorbate. As long as the island keeps sliding on a monolayer, friction increases with Γ but decreases again discontinuously as soon as a second layer appears between gold and graphite.

**Figure 3:**
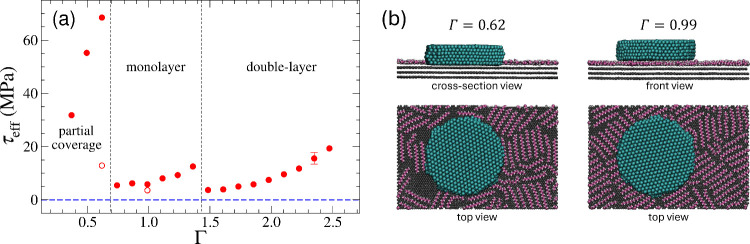
(a) Effective shear stress (τ_eff_) as a function of the nominal coverage (Γ). Black, dashed lines separate the cases where gold slides directly on graphite at small Γ, on top of a monolayer, and on top of a double-layer of *n*-hexadecane (HEX) molecules. The blue, dashed line represents the mean shear stress for clean gold-graphite contacts. Solid and hollow symbols represent results from *v* = 20 and 2 m/s sliding velocity, respectively. (b) Snapshots at different Γ at *v* = 2 m/s.

At Γ ≈ 0.62 and Γ ≈ 1, simulations were run with the velocity reduced by a factor of 10. The resulting friction decreased by factors of 5 and 2, respectively. These changes are too significant for the friction to be labeled as Coulombic, i.e., barely dependent on velocity, although the velocities exceed the experimental ones by 9 orders of magnitude. It is worth noting that the trailing edge of a Γ = 0.62 contact drags along some polymers at *v* = 2 m/s but not at *v* = 20 m/s. Without this effect, the friction-velocity relation would have moved closer to a linear, i.e., Stokesian dependence.

Since the importance of the area below the island relative to that of the contact line or circumference is larger in the experiments than in the simulations (roughly by a factor of 10, because the experimental radii exceed the simulated ones by that factor), we also simulated selected area-filling contacts between gold and graphite (Figure 4). For this purpose, we explored two coverages, Γ = 1 and Γ = 6, as well as two relative orientations between the graphite and the Au (111) surfaces so that the Au surface is ideally aligned with graphite, i.e.,  is aligned with the graphite armchair direction, Gr_A_, albeit with a lattice mismatch of roughly 17% and one time misoriented by 90° so that  is parallel to Gr_A_. For both orientations, the true areal shear stress at Γ = 1 is rather small compared to the effective shear stress of ≲6 MPa for the island, i.e., 0.26 MPa for the  orientation and 1.2 MPa for . At Γ = 6, shear stress changes to τ ≈ 0.87 MPa. Results are shown in Figure 4a.

**Figure 4:**
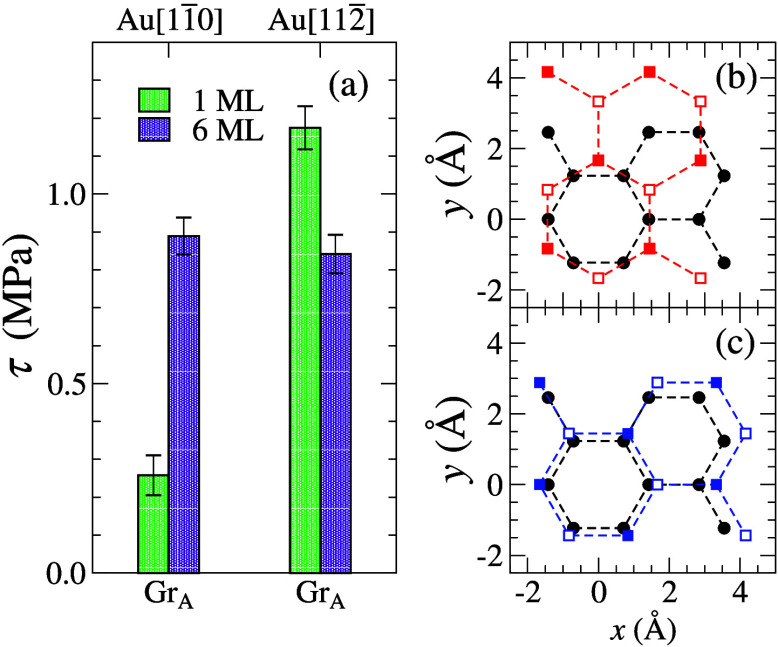
(a) Shear stress (τ) between two area-filling graphite and gold solids separated by either a single- (1 ML) or a six-monolayer (6 ML) HEX film. The top and bottom ticks indicate the slab material and lattice orientation with the armchair direction of graphite (Gr_A_) being aligned with the sliding velocity **v** = 10 **e**_*x*_ m/s. In-plane projections of (b) Au  (red squares) and (c) Au  (blue squares) on Gr_A_ (black circles). Solid and hollow squares indicate Au atom positions in two adjacent (111) layers, respectively.

These results are interesting for a variety of reasons. First, they show that relative orientation can matter even between (quasi-) incommensurate solids as τ differs by a factor of almost five between the two configurations at Γ = 1. Thus, for any system, in which Γ is small and areal friction dominates, large scatter in the data is unavoidable. Second, the concept of continuum mechanics is far from being applicable in our system, as a 6-fold thicker lubricant can yield a 3-fold increase in friction, at least for the misaligned surfaces, while continuum-based concepts, in particular those based on Reynolds’ thin-film equation, would expect it to drop with increasing Γ. Third and perhaps most importantly for our present purposes, friction for the simulated islands is indeed dominated by processes outside the contact, since their effective shear stress was found to be 5.8 MPa at 20 m/s and 3.0 MPa at 2 m/s each time at Γ = 1. Thus, for islands with ten times the radius, areal friction can dominate but the friction stemming from noncontact is not yet necessarily negligible. In fact, when the mean areal stress decreases with contact area, as is the case for structurally lubric contacts, the noncontact friction may well be dominant, in particular at the small (or zero) normal loads studied in this work. Having provided expectations from atomistic simulations, we return to the presentation and interpretation of experimental results.

Previous studies show that a freshly prepared graphite surface becomes completely coated with molecular contaminants in just a few minutes when exposed to air.^32^ Thus, considering that our experiments are conducted under uncontrolled, ambient conditions, some surface contamination will be present, even if it does not reveal itself in AFM images. This is why we classify a sample as *lightly contaminated* even if the contamination is not revealed by AFM. However, when contamination is clearly visible in phase and topography images, which typically happens >1 week after synthesis, we deem the sample to be *heavily contaminated*. To explore the impact of the degree of contamination on aging, rejuvenation, and friction switches, we repeated our nanomanipulation experiments for *heavily contaminated* islands. Table 1 reports the results. The numbers expressed under “frac.” (short for fraction) contain the number of experiments in the denominator. The fractions are converted to percentages for clearer comprehension. Remarkably, we observe that increased levels of contamination significantly diminish the observation frequency of all three effects (rejuvenation, aging, and friction switches), by 75% and more.

**Table 1:** Comparative Analysis of Observation Frequencies of the Three Effects for Lightly Contaminated and Heavily Contaminated Islands

	Degree of *Rejuvenation*		Degree of *Aging*		Degree of *Switches*	
Contamination	frac.	%	frac.	%	frac.	%
light	26/26	100	8/8	100	15/26	58
heavy	20/82	24	5/22	23	6/82	7

While all islands in lightly contaminated samples exhibit the rejuvenation and aging effects, and more than half the switch effect (with friction between the two branches differing by factors from three to ten), the majority of heavily contaminated islands show consistent friction levels across all manipulations. The associated shear stresses align more closely with the low-friction branch of the lightly contaminated islands, with the exception of a few data points, as can be seen in Figure 5, which shows shear stresses as a function of contact area*A*. Please note that Figure 5 specifically contains data for lightly contaminated islands that exhibit the switch effect (15), and for heavily contaminated islands that do not exhibit any of the three effects (55). All three cases, i.e., heavy contamination as well as high and low friction branches at light contamination, reveal the trend that larger islands have smaller shear stresses. This observation can be a sign of structural lubricity and/or a friction mechanism that is dominated by the contact line. Surprisingly, the trend is most pronounced in the case of heavy contamination, where τ evolves almost linearly with 1/*A*, at least for *A* ≲ 15,000 nm^2^. Such a strong scaling of the shear stress does not appear plausible outside the realm of structural lubricity.

**Figure 5:**
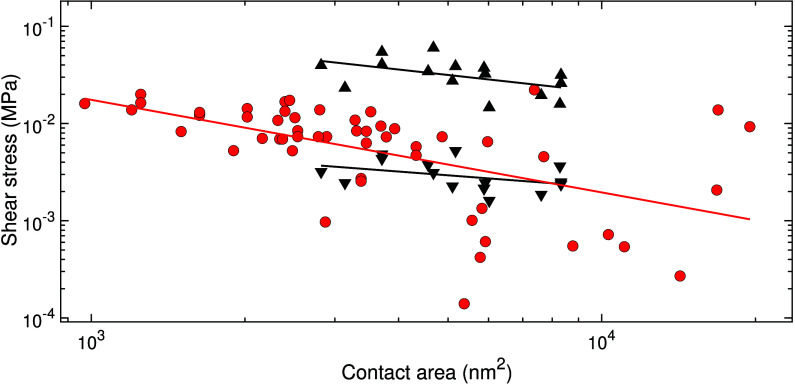
Shear stress as a function of contact area, experimentally recorded for lightly contaminated and heavily contaminated islands. Black triangles represent shear stresses associated with lightly contaminated islands that exhibit the switch effect, separated into two branches (upward triangles for the high friction branch, downward triangles for the low). Red circles represent shear stresses associated with heavily contaminated islands that do not exhibit any of the three effects. The error in contact areas due to tip-convolution effects is estimated to be at ±10%.^33^

In summary, simulations and experiments reveal a nonmonotonic relationship between friction and exposure time or coverage, respectively. Correlating these remains speculative due to simulations being conducted at relatively small island sizes and high velocities, favoring large shear stress. Nevertheless, several conclusions can be drawn. First, direct contact between graphite and nanoislands is unlikely to be relevant for our observations. In principle, dislocations and structural defects in the islands can also lead to changes in friction levels.^34−36^ However, shear stresses in the experiments are rather below than above those in the simulations so that we do not see the need to introduce additional effects into the simulations that would further increase that gap. Moreover, during the initial stages of our simulations, i.e., before introducing contamination, we explored gold clusters with point and line defects. Yet, friction remained much below that observed with contamination. Potentially more importantly, the von Mises yield strength of gold at the macroscopic scale is on the order of 100 MPa, while experimental shear stresses are clearly less than 0.1 MPa at the nanoscale, at which point introducing dislocations becomes difficult or rather impossible. Lastly, the mild/vanishing load conditions in the experiments preclude the possibility of friction-enhancing structural defects in graphite. Second, different friction mechanisms must be at work for different levels of contamination. If friction was dominated by viscous or plastic processes in the “boundary lubricant” (i.e., “in-contact”), shear stress would not decrease as rapidly with island size in any regime, i.e., roughly with 1/*A*^0.6^ and 1/*A*^0.4^ in the high- and low-friction branch of light contamination, respectively, and most surprisingly with almost 1/*A* in the heavily contaminated regime. However, the large scatter of individual measurements at a given island size suggests orientational effects between gold and graphite, as predicted by the simulations. Third, and perhaps most interestingly, contacts remain superlubric despite contamination. This conclusion finally provides an answer as to why ultralow friction was observed experimentally for the gold-graphite material system under ambient conditions.^13^ The underlying mechanism observed in the simulations is that the adsorbates form a locally commensurate structure with graphite, whereby slip happens at the adsorbate-gold interface. It should also be noted that the high friction recorded during the first areal scan is most likely caused by the “plowing” of molecular contaminants out of the scan area and out-of-contact conformational changes in the adsorbed molecular layer, as suggested by the simulations (see the high shear stresses in the ”partial coverage” regime shown in Figure 3a). Subsequent scans involve no displacement of adsorbates on the now-swept area, resulting in low friction.

Finally, we note that high- and low-friction branches observed in our experiments differ from previously observed “frictional duality”, which was related to the presence and complete absence of contamination,^12^ and not differing degrees of contamination. Our observation that structural lubricity of graphite can “survive” contamination down to minute scales aligns with the recently observed ultralow friction exhibited by turbostratic graphite under ambient conditions.^37,38^

## Supporting Information Available

Experimental methods and materials, molecular dynamics simulations, figures describing sample topography, force–distance spectroscopy, the “tip-on-top” manipulation method, model system for molecular dynamics simulations, observation of all three effects on the same island, and dynamic nature as well as desorption of the contamination layer (PDF)
