# Supplementary material for: Persistence of Structural Lubricity on Contaminated Graphite: Rejuvenation, Aging, and Friction Switches
Source: Nano Lett. 2024 Sep 23;24(39):12118–24. doi: 10.1021/acs.nanolett.4c02883 (PMC11450813; doi:10.1021/acs.nanolett.4c02883)
Supplement: Supplementary file 1 — nl4c02883_si_001.pdf [file nl4c02883_si_001.pdf]

Supporting Information

Persistence of Structural Lubricity on Contaminated Graphite: Rejuvenation, Aging, and Friction Switches

Wai H. Oo,[†,¶] Hongyu Gao,[*,‡,¶] Martin H. Müser,[‡] and Mehmet Z. Baykara[*,†]

†*Department of Mechanical Engineering, University of California Merced, Merced, California 95343, USA*

‡*Department of Materials Science and Engineering, Saarland University, Saarbrücken, Saarland 66123, Germany*

¶*These authors contributed equally to this work*

E-mail: hongyu.gao@uni-saarland.de; mehmet.baykara@ucmerced.edu

Experimental Methods and Materials

The samples studied here were synthesized by the evaporation of 99.999% purity gold (∼2 nm) on freshly cleaved highly oriented pyrolytic graphite (HOPG) surfaces under high vacuum conditions (∼10^{-8} Torr), at an elevated substrate temperature in the range of 200 – 250°C. The deposition rate, deposition time, and the temperature during the evaporation can be adjusted to create islands with varying size distributions. This process yields a sample system where crystalline gold nanoislands, with mostly straight edges, decorate the atomically flat graphite surface (Figure S1). The structural and morphological details of the sample system were analyzed via atomic force microscopy and electron microscopy in previous work.[1,2] While most islands are arranged along the step edges of the graphite substrate, some can be found on flat terraces away from the edges, which are the ones that are utilized for the manipulation experiments reported here. All experiments were performed with a commercial AFM (Asylum Research, Cypher VRS) under uncontrolled ambient conditions (temperatures at 30-35°C, and humidity levels at 30-40%) and with commercial cantilevers (Asylum Research, ASYELEC.01-R2), which were calibrated for both normal and lateral force measurements.[3,4] For the cantilevers used in our experiments, the lateral calibration factors were, on average, 825 nN/V, and normal spring constants, 2.8 N/m. The particular choice of cantilevers was mainly motivated by their compatibility with both tapping and contact mode AFM, which were required for the topographical imaging (tapping mode) and nanomanipulation (contact mode) experiments. Additional experiments performed with another type of cantilever (Budget Sensors, Tap150Al-G, with average normal spring constants of 5 N/m) yielded similar results. Overall, the success rate of our manipulation attempts was about 90%.

While our tip-on-top manipulation approach shares fundamental similarities with the methodology utilized by Dietzel *et al.*[5] in their nanomanipulation studies, there are noteworthy distinctions. In our experiments, no external normal load is imposed on the island during the manipulation process, because the contact between the tip and the island solely

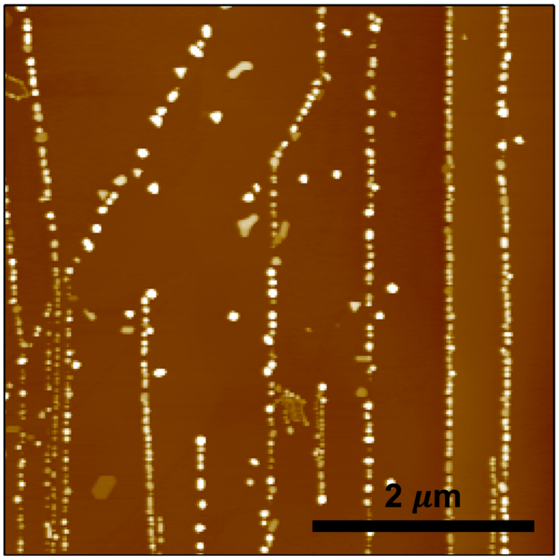

Figure S1: AFM topography image of gold nanoislands on the graphite substrate. Islands located at step edges form lines. Isolated islands on terraces are those being manipulated.

relies on adhesive interaction forces (<10 nN, Figure S2). Owing to the superlubric nature of the island-substrate interface, the static friction between the gold island and the graphite substrate is significantly lower than that between the tip apex and the island. Consequently, as soon as a contact mode scan commences on the island surface, the island begins sliding along with the tip. Since there is no substantial relative motion between the tip and the island, we record a "flat" topography (Figure S3b) and the lateral force channel captures the lateral forces acting at the sliding interface between the island and the substrate. By comparing scans before and after the manipulation, as shown in Figure S3c,d, we observe that the islands often rotate from their initial registries. These rotations may occur because (i) the tip might not be directly on top of the island's center of mass, causing torque, or (ii) the tip may occasionally slip from the island during the manipulation. Together with complementary molecular dynamics simulations, we investigated the role of rotations in tip-on-top manipulations in an earlier paper[6] and found no evidence that the rotations would result in the specific effects of rejuvenation, aging and switches we observe here, despite previous reports of rotation-induced breakdown of superlubricity in graphite-graphite contacts.[7] The second major difference between our work and earlier ones is that the island is continuously

manipulated by the tip over the duration of an entire scan consisting of multiple scan lines, as opposed to collecting data over a single scan line.[5] In particular, during manipulation, the AFM tip drags the island on the graphite substrate in a raster scan pattern. For example, during a 30×30 nm^2 scan consisting of 256 lines, the island is moved forwards and backwards by 30 nm, 256 times in each direction. Compared to previous work,[2,5,8,9] this approach increases the amount of friction force data collected drastically, thereby resulting in improved reliability. After a few hours of operation, lateral thermal drift rates associated with our system were found to be on the order of a few nanometers per scan, thus not leading to complications in our data acquisition procedure. In order to further test the repeatability of our findings, scans were performed multiple times (typically five or more) on a given island. This repeated data collection resulted in new findings, which are reported in the manuscript.

It is important to note that an analysis of individual lateral force scans during island manipulation (such as those in Fig. 1d) reveals signal fluctuations which manifest as standard deviation values on the order of several tens of pN. This shows that the mean friction forces recorded in the low friction regime are on the same order of magnitude as, and somewhat smaller than, standard deviation values resulting from noise. This is perhaps not surprising as structural lubricity (in the absence of effects such as molecular plowing relevant for the high friction regime) is expected to lead to "nearly vanishing" friction forces. Most importantly, however, data in Fig. 5 show that shear stresses scale negatively with contact area, a clear signature of structural lubricity that is not found in non-structurally-lubric systems. This unequivocally shows that the experimentally recorded friction data in the low friction regime carry physical significance and do not simply result from noise.

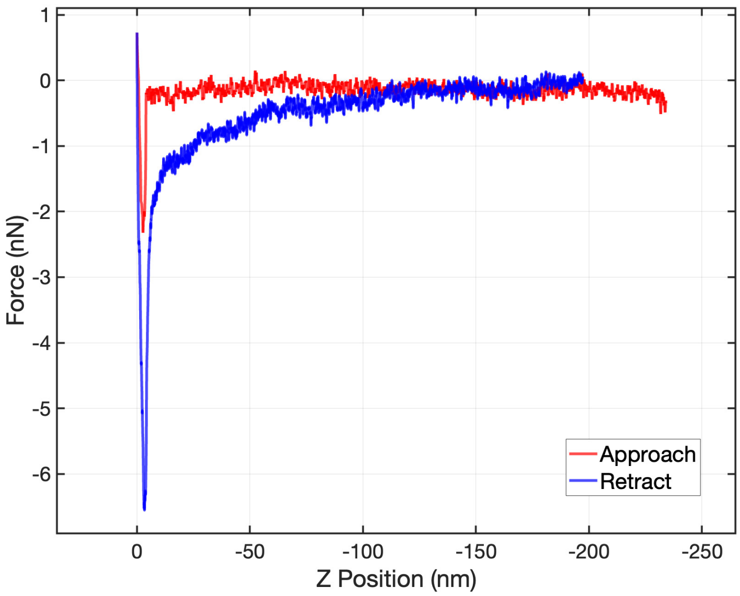

Figure S2: Force-distance curve taken on top of a gold island surface. Adhesion is ∼6.5 nN.

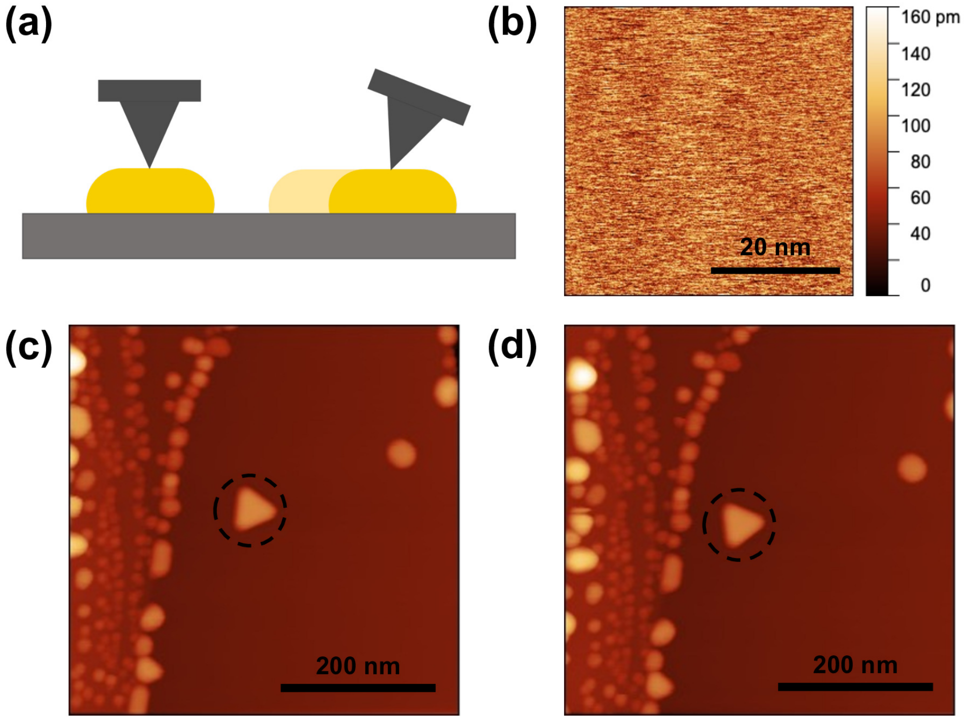

Figure S3: (a) Schematic showing the "tip-on-top" island manipulation method. (b) Topography map during island manipulation process shows no discernible contrast, proving that the tip is dragging the island laterally instead of scanning over its surface. (c, d) Tapping-mode topography images before and after an island manipulation, showing that the island was displaced from its initial position.

Molecular Dynamics Simulations

A circular gold cluster with a (111) surface sliding on HOPG adsorbed with n-hexadecane (HEX) is modeled using MD simulations as sketched in Figure S4. The cluster is connected to a spring moving at a constant speed of 2–20 m/s. The in-plane dimensions of HOPG are 11.5×7.4 nm^2, with periodic boundary conditions applied. The radius of the cluster is approximately 2.85 nm. The number of HEX molecules, $30 \leq n_{\mathrm{HEX}} \leq 200$, results in partial coverages from $\Gamma \approx 0.4$ to 2.5 monolayers. The cluster slides under its own adhesive load without constraints on its rotational degrees of freedom. The temperature is maintained at 300 K using a Langevin thermostat applied to the mid-layer of the HOPG, while the bottom layer of HOPG is kept at zero stress. Inter- and intra-molecular interactions of HEX are described using the L-OPLS-AA force-field.[10,11] Individual interactions for gold and graphite are described by an EAM potential[12] and the AIREBO potential,[13] respectively. Cross interactions between gold and graphite are described by a Morse potential.[14] All other cross interactions are described using a 12-6 Lennard-Jones potential with parameters[15–17] converted according to the Lorentz-Berthelot mixing rules.[18,19] All MD simulations are carried out using the open-source software LAMMPS.[20]

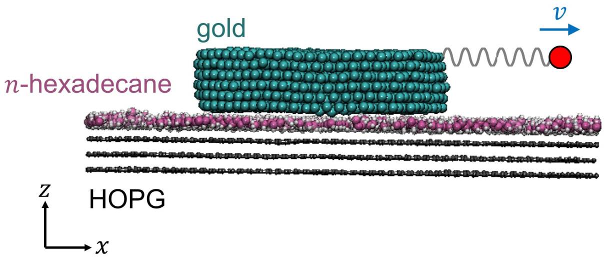

Figure S4: Snapshot of the MD model system before sliding. The gold cluster's center-of-mass is connected to a spring of stiffness 160 N/m. There are 50 HEX molecules in this snapshot, corresponding to a coverage of approximately 0.6 monolayers.

Supplementary Figures

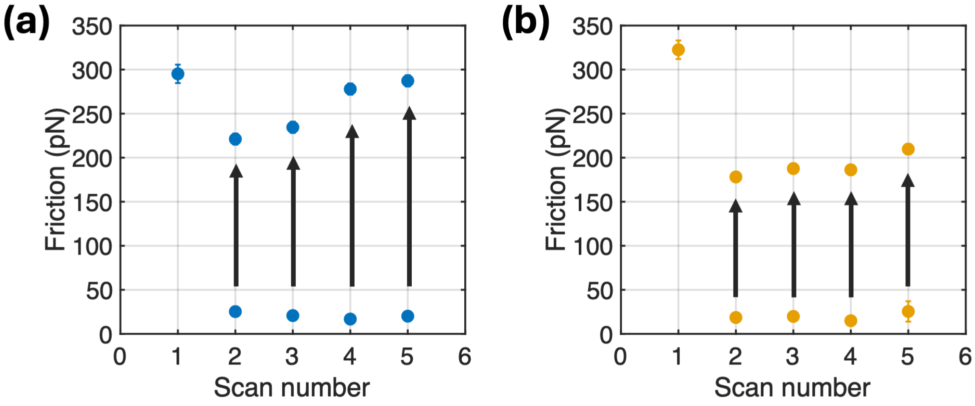

Figure S5: Observation of all three effects on the same nanoisland. (a) Friction force as a function of scan number extracted from the nanomanipulation of a freshly deposited island. (b) Same as (a) but repeated on the same island and area after a 30 minute waiting time.

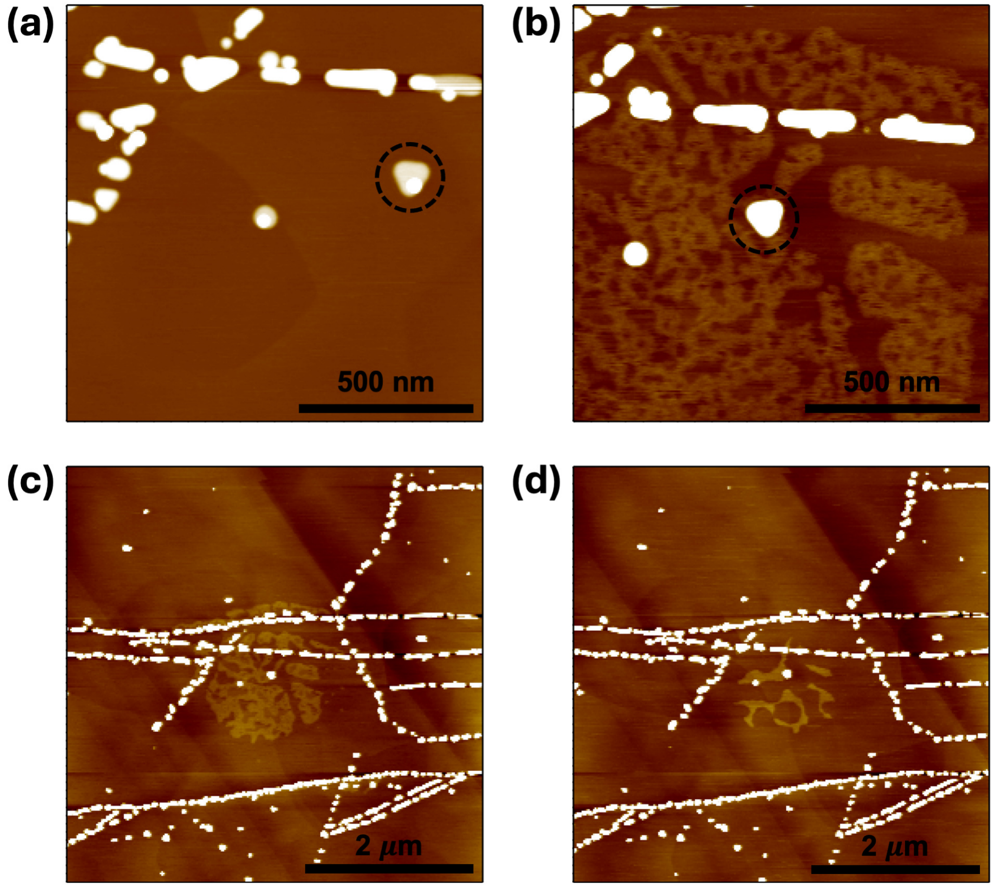

Figure S6: Dynamic nature of the contamination layer on a heavily contaminated sample. (a) AFM topography image showing an island and the adjacent topography before manipulation, recorded on a 4-month-old sample. Contamination is not yet evident in this image, perhaps due to the even distribution of the contamination layer on the entire sample surface. (b) AFM topography image showing clear signs of contamination (about 1 nm thick) after the highlighted island was manipulated. (c) Large-scale AFM topography image, zoomed out from (b). (d) Large-scale topography image of the same area in (c), after the sample was left overnight in the AFM for 14 hours.

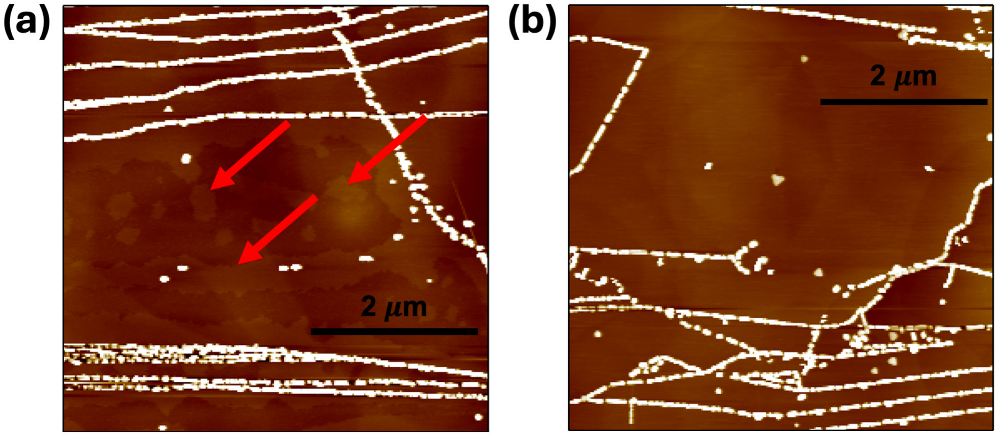

Figure S7: Desorption of the contamination layer after heating. (a) AFM topography image showing significant contamination (partially highlighted by red arrows) on a 4-month-old sample, at 33°C. (b) AFM topography image of the same sample after heating up to 100°C, with no evidence of the contamination layer.

References

(1) Cihan, E.; Özoğul, A.; Baykara, M. Z. Structure and nanotribology of thermally deposited gold nanoparticles on graphite. *Applied Surface Science* **2015**, *354*, 429–436.

(2) Cihan, E.; İpek, S.; Durgun, E.; Baykara, M. Z. Structural lubricity under ambient conditions. *Nature Communications* **2016**, *7*, 12055.

(3) Sader, J. E.; Chon, J. W. M.; Mulvaney, P. Calibration of rectangular atomic force microscope cantilevers. *Review of Scientific Instruments* **1999**, *70*, 3967–3969.

(4) Varenberg, M.; Etsion, I.; Halperin, G. An improved wedge calibration method for lateral force in atomic force microscopy. *Review of Scientific Instruments* **2003**, *74*, 3362–3367.

(5) Dietzel, D.; Feldmann, M.; Fuchs, H.; Schwarz, U. D.; Schirmeisen, A. Transition from static to kinetic friction of metallic nanoparticles. *Applied Physics Letters* **2009**, *95*, 053104.

(6) Oo, W.; Baykara, M.; Gao, H. A Computational Study of Cluster Dynamics in Structural Lubricity: Role of Cluster Rotation. *Tribology Letters* **2023**, *71*, 115.

(7) Filippov, A. E.; Dienwiebel, M.; Frenken, J. W. M.; Klafter, J.; Urbakh, M. Torque and Twist against Superlubricity. *Phys. Rev. Lett.* **2008**, *100*, 046102.

(8) Özoğul, A.; İpek, S.; Durgun, E.; Baykara, M. Z. Structural superlubricity of platinum on graphite under ambient conditions: The effects of chemistry and geometry. *Applied Physics Letters* **2017**, *111*, 211602.

(9) Dietzel, D.; Ritter, C.; Mönninghoff, T.; Fuchs, H.; Schirmeisen, A.; Schwarz, U. D. Frictional Duality Observed during Nanoparticle Sliding. *Phys. Rev. Lett.* **2008**, *101*, 125505.

(10) Price, M. L. P.; Ostrovsky, D.; Jorgensen, W. L. Gas-phase and liquid-state properties of esters, nitriles, and nitro compounds with the OPLS-AA force field. *Journal of Computational Chemistry* **2001**, *22*, 1340–1352.

(11) Siu, S. W. I.; Pluhackova, K.; Böckmann, R. A. Optimization of the OPLS-AA Force Field for Long Hydrocarbons. *Journal of Chemical Theory and Computation* **2012**, *8*, 1459–1470, PMID: 26596756.

(12) Zhou, X. W.; Johnson, R. A.; Wadley, H. N. G. Misfit-energy-increasing dislocations in vapor-deposited CoFe/NiFe multilayers. *Phys. Rev. B* **2004**, *69*, 144113.

(13) Stuart, S. J.; Tutein, A. B.; Harrison, J. A. A reactive potential for hydrocarbons with intermolecular interactions. *The Journal of Chemical Physics* **2000**, *112*, 6472–6486.

(14) de la Rosa-Abad, J. A.; Soldano, G. J.; Mejía-Rosales, S. J.; Mariscal, M. M. Immobilization of Au nanoparticles on graphite tunnels through nanocapillarity. *RSC Adv.* **2016**, *6*, 77195–77200.

(15) Jorgensen, W. L.; Maxwell, D. S.; Tirado-Rives, J. Development and Testing of the OPLS All-Atom Force Field on Conformational Energetics and Properties of Organic Liquids. *Journal of the American Chemical Society* **1996**, *118*, 11225–11236.

(16) Pu, Q.; Leng, Y.; Zhao, X.; Cummings, P. T. Molecular simulations of stretching gold nanowires in solvents. *Nanotechnology* **2007**, *18*, 424007.

(17) Pykal, M.; Jurečka, P.; Karlický, F.; Otyepka, M. Modelling of graphene functionalization. *Phys. Chem. Chem. Phys.* **2016**, *18*, 6351–6372.

(18) Lorentz, H. A. Ueber die Anwendung des Satzes vom Virial in der kinetischen Theorie der Gase. *Annalen der Physik* **1881**, *248*, 127–136.

(19) Berthelot, D. Sur le mélange des gaz. *Compt. Rendus* **1898**, *126*, 15.

(20) Plimpton, S. Fast Parallel Algorithms for Short-Range Molecular Dynamics. *Journal of Computational Physics* **1995**, *117*, 1–19.
